# Disease Tolerance Mediated by Phosphorylated Indoleamine-2,3 Dioxygenase Confers Resistance to a Primary Fungal Pathogen

**DOI:** 10.3389/fimmu.2017.01522

**Published:** 2017-11-13

**Authors:** Eliseu Frank de Araújo, Flávio Vieira Loures, Cláudia Feriotti, Tania Costa, Carmine Vacca, Paolo Puccetti, Luigina Romani, Vera Lúcia Garcia Calich

**Affiliations:** ^1^Department of Immunology, Institute of Biomedical Sciences, University of São Paulo, São Paulo, Brazil; ^2^Department of Experimental Medicine, University of Perugia, Perugia, Italy

**Keywords:** paracoccidioidomycosis, indoleamine-2,3 dioxygenase, pulmonary dendritic cells, disease tolerance, phosphorylated indoleamine-2,3 dioxygenase, signaling function

## Abstract

Resistance to primary fungal pathogens is usually attributed to the proinflammatory mechanisms of immunity conferred by interferon-γ activation of phagocytes that control microbial growth, whereas susceptibility is attributed to anti-inflammatory responses that deactivate immunity. This study challenges this paradigm by demonstrating that resistance to a primary fungal pathogen such as *Paracoccidiodes brasiliensis* can be mediated by disease tolerance, a mechanism that preserves host fitness instead of pathogen clearance. Among the mechanisms of disease tolerance described, a crucial role has been ascribed to the enzyme indoleamine-2,3 dioxygenase (IDO) that concomitantly controls pathogen growth by limiting tryptophan availability and reduces tissue damage by decreasing the inflammatory process. Here, we demonstrated in a pulmonary model of paracoccidioidomycosis that IDO exerts a dual function depending on the resistant pattern of hosts. IDO activity is predominantly enzymatic and induced by IFN-γ signaling in the pulmonary dendritic cells (DCs) from infected susceptible (B10.A) mice, whereas phosphorylated IDO (pIDO) triggered by TGF-β activation of DCs functions as a signaling molecule in resistant mice. IFN-γ signaling activates the canonical pathway of NF-κB that promotes a proinflammatory phenotype in B10.A DCs that control fungal growth but ultimately suppress T cell responses. In contrast, in A/J DCs IDO promotes a tolerogenic phenotype that conditions a sustained synthesis of TGF-β and expansion of regulatory T cells that avoid excessive inflammation and tissue damage contributing to host fitness. Therefore, susceptibility is unexpectedly mediated by mechanisms of proinflammatory immunity that are usually associated with resistance, whereas genetic resistance is based on mechanisms of disease tolerance mediated by pIDO, a phenomenon never described in the protective immunity against primary fungal pathogens.

## Introduction

Indolamine-2,3 dioxygenase (IDO) is an intracellular enzyme of crucial importance in the tryptophan (trp) catabolism acting along the kynurenine (Kyn) pathway (1, 2). Although the cytokine IFN-γ is considered the major inducer of IDO expression, TGF-β, TNF-α, prostaglandins, and toll-like receptor ligands, among others, have also the ability of promoting the expression of the IDO gene (3–5). Among the cells of myeloid origin, dendritic cells (DCs) express the highest levels of IDO that is, however, also present in macrophages, neutrophils, endothelial cells, and fibroblasts (6–8).

Indolamine-2,3 dioxygenase was primarily associated with host defense against pathogens due to trp starvation caused by its enzymatic activity (9–11). However, the recent recognition of the immunoregulatory function of IDO and Kyn, put IDO to another level of knowledge and relevance (11, 12). Indeed, IDO has been recently described as an intracellular signaling molecule involved in the sustained tolerogenic activity of DCs. This signaling function is mediated by TGF-β signaling that promotes IDO phosphorylation that ultimately activates the non-canonical NF-κB pathway that triggers genes encoding IDO, TGF-β, IFN-α, and IFN-β, that maintain the tolerogenic activity of murine plasmacytoid DCs (12). This likely accounts for the ability of IDO to favor microbial persistence and concomitant control of inflammation during chronic infections (13–15). Therefore, acting *via* trp depletion and Kyn production, IDO can inhibit the proliferation of T cells and induce their apoptosis. Furthermore, through the aryl hydrocarbon receptor (AhR), IDO directs the conversion of naive CD4^+^ T cells into regulatory Foxp3^+^ T cells (Tregs) (13, 16, 17).

Paracoccidioidomycosis (PCM), is a granulomatous disease caused by two species of the dimorphic fungus *Paracoccidioides, P. brasilienis*, and *P. lutzii* (18). Immunoprotection to PCM is mediated by prevalent Th1/Th17 immunity, whereas Th2 and Th9 responses are associated with severe forms of the disease (19–21). In a murine model of pulmonary infection, A/J and B10.A mice were described, respectively, as resistant and susceptible to PCM. The A/J mouse strain develops a chronic and regressive PCM restricted to the lungs. Its adaptive immunity is mainly mediated by Th1 and Th17 cells that are tightly controlled by elevated numbers and activity of Treg cells. In contrast, B10.A mice develop a progressive disseminated disease associated with large amounts of fungi in non-organized lesions that mimic the severe forms of PCM (22–27).

The immunoregulatory mechanisms that control resistance to PCM are complex and not yet completely solved. In this aspect, our previous studies have shown that in pulmonary PCM, IDO is an important immunoregulatory enzyme that promotes fungal clearance and inhibits T cell immunity but only in susceptible mice IDO inhibition by 1-methyl-dl-tryptophan (1MT) caused progressive tissue pathology and increased mortality rates (28). This difference appeared to be mediated by the opposite innate immunity of resistant and susceptible mice. Indeed, in B10.A mice the innate immunity is preferentially proinflammatory with elevated production of IL-12 and IFN-γ, whereas in A/J mice the predominant TGF-β secretion provides an anti-inflammatory innate response (26–30). These findings led us to hypothesize that IDO has distinct immunoregulatory roles in pulmonary PCM. Here, we could demonstrate that in susceptible mice IDO activity is IFN-γ-dependent and mediated by its catalytic activity, whereas in resistant mice a prevalent TGF-β signaling triggered IDO phosphorylation imparting a signaling and tolerogenic function. The TGF-β-IDO-Treg interplay generates an early pathogen tolerance that allows A/J mice to interact with a primary fungal pathogen as a commensal microbe. Thus, the signaling function of pIDO may lead to an unusual fungus–host interaction that efficiently balances tolerance and resistance mechanisms to the benefit both the pathogen and the host.

## Materials and Methods

### Mice

Susceptible (B10.A) and resistant (A/J) mouse strains to *Paracoccidiodes brasiliensis* infection were obtained from our Isogenic Unit (Immunology Department of Institute of Biomedical Sciences of University of São Paulo, Brazil) and used at 8–11 weeks of age. Specific pathogen free mice were fed with sterilized laboratory chow and water *ad libitum*.

### Fungus

*Paracoccidiodes brasiliensis* 18 (Pb 18) was used throughout this investigation. To ensure the maintenance of its virulence, the isolate was used after three serial animal passages. Pb18 yeast cells were maintained by weekly sub cultivation in semisolid Fava Netto culture medium at 37°C and used on the seventh day of culture. Phosphate-buffered saline (PBS)-washed yeast cells were adjusted to 20 × 10^6^ cells/mL based on hemocytometer counts. Viability was determined with Janus Green B vital dye (Merck) and was always higher than 85%.

### 1MT Treatment and *In Vivo* Fungal Infection

Mice were anesthetized and submitted to intratracheal (i.t.) *P. brasiliensis* infection as previously described. Briefly, after intraperitoneal (i.p.) anesthesia the animals were infected with 1 × 10^6^ Pb18 yeast cells, contained in 50 µL of PBS, by surgical i.t. inoculation, which allowed dispensing of the fungal cells directly into the lungs. The skins of the animals were then sutured, and the mice were allowed to recover under a heat lamp. Groups of infected B10.A and A/J mice were treated with daily i.p. injections of 5 mg/mL of 1MT or 1 mg/mL of rice starch (Sigma-Aldrich) as control. Mice were sacrificed after 96 h or 2 weeks of infection.

### Isolation of Pulmonary CD11c^+^ Cells

Cell suspensions of the lungs were prepared as previously described (31). The lungs were removed and digested for 60 min in RPMI-1640 medium (Sigma) containing collagenase (2 mg/mL) and DNAse (30 mg/mL). The erythrocytes were lysed with lysis buffer (TRIS + ammonium chloride) and viability determined by the trypan blue exclusion test with viabilities at least to 95%. CD11c^+^ cells were isolated from total pulmonary cells by magnetic microbeads (Miltenyi Biotec, Cologne, Germany). Pellets of pulmonary cells were counted, washed and the pellet resuspended in 400 µL of buffer (PBS + 0.5% BSA + 2 mM EDTA). The Trypan Blue dye exclusion test was used to determine the number of viable cells present in the DCs cell suspension with viabilities at least to 95%. Then, 100 µL of anti-CD11c coated microspheres were added for each 10^8^ cells and incubated for 15 min at 2–8°C. The cells were washed, the supernatant removed and the pellet resuspended in 500 µL of buffer. This suspension was fractionated using a magnetic separation column, and purified CD11c^+^ DCs obtained. This fractionation methods resulted in 95% CD11c^+^ DCs.

### Culture of DCs

The viability of the CD11c^+^ cells was evaluated by the trypan blue exclusion test, and was always higher than 85%. The cell suspensions were then centrifuged at 1,200 rpm at 4°C for 10 min and resuspended in 1.0 mL of culture medium (RPMI-1640 medium) supplemented with 10% fetal bovine serum. The cells were adjusted to 1 × 10^6^/mL and 500 µL were dispensed into each well of 24-well culture plates. The cultures were incubated at 37°C in an incubator containing 5% CO_2_–95% air for 24 h. After this period, the cells were removed and used in different assays.

### CFU Assay

After 24 h of culture, DCs suspensions were lysed and 100 µL of pellets plated onto BHI-agar medium containing 5% “fungus growth factor” and 4% horse serum (32). The plates were incubated at 35°C and colonies counted daily until no increase in CFU was observed. The numbers (log10) of viable *P. brasiliensis* colonies are expressed as the mean ± SE. Two to three experiments were performed separately.

### NO and Cytokines Measurement

Supernatants from DC cultures and lymphoproliferation assays were separated and stored at −70°C. The levels of IL-12, IL-1β, TGF-β, IL-6, IFN-γ, and IL-12 were measured by a capture enzyme-linked immunosorbent assay (ELISA) with antibody pairs purchased from eBioscience. The ELISA procedure was performed according to the manufacturer’s protocol, and absorbance was measured with a Versa Max Microplate Reader (Molecular Devices). The concentrations of cytokines were determined based on a standard curve of serial twofold dilutions of murine recombinant cytokines. Nitric oxide production was quantified by the accumulation of nitrite in the supernatants from *in vitro* protocols by a standard Griess reaction (33). All determinations were performed in duplicate, and results were expressed as micromolar concentration of NO.

### Determination of IDO Enzymatic Activity

To monitor IDO enzymatic activity, Kyn were measured using a modified spectrophotometric assay (5). The amount of 50 µL of 30% trichloroacetic acid was added to 100 µL of DCs supernatants, vortexed, and centrifuged at 800 *g* for 5 min. A volume of 75 µL of the supernatant was then added to an equal volume of Ehrlich reagent (100 mg P-dimethylbenzaldehyde, 5 mL glacial acetic acid) in a 96-well microtiter plate. Optical density was measured at 492 nm, using a Multiskan MS (Labsystems) microplate reader. A standard curve of defined l-Kyn concentrations (0–100 mM) was used to determine unknown Kyn concentrations.

### Flow Cytometry

Pulmonary CD11c^+^ cells were obtained from 1MT treated and untreated B10.A and A/J mice 96 h and 2 weeks after *P. brasiliensis* infection. For cell surface staining, pulmonary CD11c^+^ cells were washed and resuspended at a concentration of 1 × 10^6^ cells/mL in staining buffer (PBS 1×, 2% FBS, 0.5% NaN_3_). Fc receptors were blocked by the addition of unlabeled anti-CD16/32 (Fc block; BD Biosciences). The DCs cells were then stained for 30 min at 4°C with the optimal dilution of each labeled antibody [phycoerythrin tandem Cy7 (PE-Cy7)-labeled anti-CD11c, phycoerythrin tandem Cy5 (PE-Cy5)-labeled anti-B220, pacific blue (PB)-labeled anti-CD8, phycoerythrin (PE)-labeled anti-CD86, allophycocyanin (APC)-labeled anti-CD40, fluorescein isothiocyanate (FITC)-labeled MHCII (IA^K^), and allophycocyanin tandem Cy7 (APC-Cy7)-labeled anti-CD11b] monoclonal antibodies (mAbs) from BD Biociences. Cells were washed twice with staining buffer, fixed with 2% paraformaldehyde (Sigma), and acquired using a FACSCanto II equipment and FACSDiva^®^ software (BD Biosciences) and analyzed by the FlowJo software (Tree-Star, Ashland, OR, USA).

### Lymphocyte Proliferation Assay and Lymphocyte Phenotyping

Spleen lymphocytes from normal B10.A and A/J mice were resuspended (1 × 10^7^) in 1 mL PBS-BSA (0.1%) and 1 µL of carboxyfluorescein diacetate, succinimidly ester (CFSE) at a concentration of 5 mM (Molecular Probes, USA). The cells were incubated at room temperature in the dark for 15 min (shaking the tube constantly). The cells were washed two times with 10 mL of supplemented RPMI, counted and resuspended at a concentration of 1 × 10^6^/mL. In parallel, purified DCs from A/J and B10.A of uninfected and infected mice, treated and untreated with 1MT were centrifuged and adjusted to 1 × 10^6^/mL in supplemented medium. Aliquots of 100 µL of DCs were placed in a 96-well plate (U-bottom) in the presence of lymphocytes previously stained with CFSE at a ratio 1:10 (100 µL of DCs + 100 µL of lymphocytes). CFSE stained lymphocytes (treated or not with 1MT), purified DCs and Concanavalin A (Sigma) stimulated lymphocytes were used as controls. The cells were incubated at 37°C in 5% CO_2_ for 3 days. After the incubation period the cells were washed and resuspended at a concentration of 1 × 10^6^ cells/mL in staining buffer (PBS 1×, 2% FBS, 0.5% NaN_3_). Fc receptors were blocked by the addition of unlabeled anti-CD16/32 (Fc block; BD Biosciences). The leukocytes were then stained for 30 min at 4°C with the optimal dilution of each antibody labeled with the adequate fluorochrome (BD Biosciences). PE-labeled anti-CD44, PE-Cy7-labeled anti-CD8, and PerCP Cy5.5-labeled anti-CD4, and APC-Cy7-labeled anti-CD62L mAbs from BD Biosciences were used. Cells were washed twice with staining buffer, resuspended in 100 µL, and an equal volume of 2% paraformaldehyde was added to fix the cells. A minimum of 50,000 events was acquired on a FACSCanto II flow cytometer using FACSDiva^®^ and FlowJo softwares. Lymphocytes were identified on forward-scatter (FSC) and side-scatter (SSC) analysis. Gated cells were measured for CD4^+^ and CD8^+^ expression followed by CD44 expression, and cells expressing high and low levels of this molecule were gated. Gated CD44^high^ cells were then measured for expression of low levels of CD62L identifying the effector/memory CD4^+^CD44^high^CD62L^low^ and CD8^+^CD44^high^CD62L^low^ subpopulations. Gated CD44^low^ cells were then measured for the expression of high levels of CD62L identifying the naive CD4^+^CD44^low^CD62L^high^ and CD8^+^CD44^low^CD62L^high^ subpopulations (Figure S2A,B in Supplementary Material). Fifty thousand cells were acquired and the data expressed as the frequency of positive cells. The cell division index was calculated and was based on the number of CFSE^+^CD4^+^ or CFSE^+^CD8^+^ T cells found in the stimulated culture/number of CFSE^+^CD4^+^ or CFSE^+^CD8^+^ T cells in the unstimulated culture (34).

### Assay to Determine Regulatory CD4^+^CD25^+^FoxP3^+^ T Cells

Culture supernatants from lymphoproliferation assays were removed and stored at 70°C for the determination of cytokines according to the manufacturer’s protocol. The pellet cells were stained with PE-Cy7-labeled anti-CD4 and PerCP Cy5.5-labeled anti-CD25 for 30 min 4°C. Next, the cells were washed with staining buffer containing 2% fetal bovine serum, 0.1% azide, 100 µL of PBS. The permeabilization of the plasma membrane was made with the addition of 200 µL/well of Cytofix/Cytoperm (Fixation/permeabilization kit BD Biosciences^®^) at room temperature for 30 min, protected from light. After washing the cells, the nuclear membrane permeabilization was performed using 130 µL/well of solution containing 750 µL of 1× PBS, 250 µL of 4% paraformaldehyde, 5 µL Tween 20 Sigma^®^ and the cells then were incubated for 30 min at room temperature in the dark.

After washing the cells with cold PBS (1×), the nuclear transcription factor FoxP3 was stained with an anti-FoxP3 labeled specific antibody (APC-labeled anti-FoxP3) diluted 1/100 in staining buffer. The cells were incubated for 30 min at 4°C, then washed with ice-cold PBS, fixed with 2% paraformaldehyde and stored at 4°C in the dark until analyzed by flow cytometry. Gates for total lymphocytes, CD4^+^, CD4^+^CD25^+^, and finally for TCD4^+^CD25^+^FoxP3^+^ T lymphocytes were made (Figure S2C in Supplementary Material). Cells were analyzed as above described.

### Intracellular Cytokines Measurement

Dendritic cells obtained from the lungs were adjusted to 1 × 10^6^ cells and stimulated for 6 h in complete medium in the presence of 100 ng/mL phorbol 12-myristate 13-acetate, 500 ng/mL ionomycin (both from Sigma-Aldrich; Germany), and monensin (3 mM, eBioscience). Cells were subsequently labeled for surface molecules (PerCP-labeled anti-CD11c) and then treated according to the manufacturer’s protocol for intracellular staining using the Cytofix/Cytoperm kit (BD Biosciences). Cells were then stained with Alexa Fluor 488-labeled anti-IDO, PB-labeled anti-IL-10, PE-Cy7-labeled anti-TNF-α, APC-CY7-labeled anti-IL-12, and PE-labeled anti-TGF-β. Cells were washed twice with staining buffer, resuspended in 100 µL, and fixed with an equal volume of 2% paraformaldehyde. The flow cytometry data were acquired and analyzed as above described.

### Quantitative Analysis of mRNA Expression

RNA was extracted from DC cultures and the RNA concentrations were determined by spectrophotometer readings at an absorbance of 260 nm. First-strand cDNAs were synthesized from 2 µg RNA using the High Capacity RNA-to-cDNA kit (Applied Biosystems) according to the manufacturer’s instructions. Gene expression for the genes of IDO, IFN-γ, janus kinase 1 (JAK1), signal transducer and activator of transcription (STAT1), IL-6, suppressor of cytokine signaling 3 (SOCS3), nitric oxide synthase (NOS2), TGF-β, small mothers against decapentaplegic 2 and 3 (SMAD2 and SMAD3), inositol polyphosphate phosphatase (SHIP), Shp1, Sh22 (tyrosine phosphatases 1 and 2), IFN-α, IFN-β, and the endogenous gene glyceraldehyde 3-phosphate dehydrogenase (GAPDH) was characterized by the real time polymerase chain reaction technique (qPCR) (Applied Biosystems, Foster City, CA, USA). The samples were placed in microcentrifuge tubes for specific qPCR testing of samples containing 2 µL cDNA + 10 µL TaqMan PCR Master Mix (Applied Biosystems, Foster City, CA, USA) according to the following thermal profile: denaturation at 95°C for 15 s, annealing at 60°C for 15 s, and extension at 72°C for 15 s. The sequences of the primers for Indo (Mm00492506_m1), IFN-γ (Mm01168134_m1), Jak1 (Mm00600614_m1), Stat1 (Mm00439531_m1), IL-6 (Mm00446190_m1), SOCS3 (Mm00545913_s1), NOS2 (Mm00440502_m1), TGF-β1 (Mm01178820_m1), Smad2 (Mm00487530_m1), Smad3 (Mm01170760_m1), Inpp5d (Ship—Mm00494987_m1), Nr0b2 (Shp1—Mm00442278_m1), PTPN11 (Shp2—Mm00448434_m1), Ifna1 (Mm03030145_gH), Ifnb1 (Mm00439552_s1), and the gene endogenous transcripts in all cell types, GAPDH (Mm99999915_g1) were obtained from Applied Biosystems ready for testing. Probes were labeled with 6-carboxyfluorescein (FAM) at their 5′-terminal end. Data were normalized to GAPDH gene expression. *Taq*Man PCR assays were performed on a Stratagene MxP3005P QPCR System and data were developed using the MxPro QPCR software (Agilent Technologies, USA).

### NF-κB DNA-Binding ELISA-Based Assay

Nuclear extracts from DCs were obtained using the Nuclear Extract Kit (Active Motif, USA). DNA binding activity of NF-κB was measured by an ELISA-based assay using a TransAM NF-κB kit (Active Motif, USA) following manufacturer’s instructions. This assay detects binding of p65, p50, p52, and Rel-B proteins to oligonucleotides containing an NF-κB consensus-binding site immobilized onto 96-well plates by specific primary antibodies that recognize an epitope that is accessible only when NF-κB is activated and bound to its target DNA. The binding activity was quantified by spectrophotometry, the OD at 450 nm.

### Western Blot Analysis

Dendritic cells were lysed in ice-cold NP40 buffer [1% NP40, 50 mmol/L Tris–HCl (pH 7.4), 150 mmol/L NaCl, 5 mmol/L EDTA containing 5 mmol/L NaF, 2 mmol/L Na_3_VO_4_, 1 mmol/L phenylmethylsulfonyl fluoride, 5 µg/mL leupeptine, and 5 µg/mL aprotinine], supplemented with a protease inhibitor (Sigma Aldrich). After incubating for 30 min at 4°C, the samples were centrifuged, and the supernatants were kept as the NP40-soluble fraction. The pellets were resuspended in SDS buffer [2% SDS, 80 mmol/L Tris (pH 6.8), 100 mmol/L DTT, and 10% glycerol]. Protein concentration was determined using a BCA assay (Pierce), and 40–60 µg of protein was run on a polyacrylamide gel and transferred to a 0.45 µm nitrocellulose membrane (Millipore, Bedford, MA, USA). Western blotting was carried out according to a standard procedure using horseradish peroxidase–conjugated secondary antibodies (Thermo Scientific and Sigma Aldrich). mAbs used were as follows: Jak1 (1:500; Sigma-Aldrich), Stat1 (1:500; Sigma-Aldrich), IKK-β (1:1,500; Sigma-Aldrich), IDO1 (1:200; Santa Cruz Biotechnology), Smad2 (1:2,500; Sigma-Aldrich), Ship (1:2,000; Sigma-Aldrich), Shp-2 (1:1,500; Sigma-Aldrich), and IKK-α (1:500; Sigma-Aldrich). Mouse monoclonal antimouse β-tubulin antibody (1:2,000; Sigma-Aldrich) was used as an internal control. The immunoreactive proteins were visualized with ECL plus reagents (ECL Western blotting Detection Reagents; Amersham) and enhanced chemiluminescence apparatus (ImageQuant LAS 4000). Densitometry was performed using ImageQuant TL 8.1 software.

### Detection of Phosphorylated IDO1 (pIDO1) by Western Blotting

Lung extracts from susceptible and resistant mice infected with *P. brasiliensis*, treated or not with 1MT were obtained after 96 h and 2 weeks of infection and analyzed by Western Blot for detection of pIDO1. Protein concentration was determined using a BCA assay (Pierce), and 20 µg of protein in Ripa Buffer and Sample buffer 4× were heated at 100°C for 4 min, then on ice for 2 min and loaded 60 μL per well in 10% SDS PAGE, 1.5 mm. Electrophoresis was then performed by transferring the gel to the nitrocellulose membrane with the membranes incubated in blocking solution [5% non-fat dry milk, 0.1% Tween 20 in Tris buffer saline (TBS) for 1 h at room temperature on an orbital shaker]. After two washings steps with TBS-Tween 20 0.1% buffer, the membrane was incubated overnight with polyclonal rabbit antimouse pIDO (CV223 AP8), 2 μg/mL in 5% non-fat dry milk, TBS at 0.1%. The membrane was washed three times for 5 min and incubated with HRP-labeled secondary antibody (anti-rabbit HRP–Pierce) for 1 h at room temperature. The revelation was performed using the ECL chemiluminescent substrate for enzymatic activity detection of peroxidase (HRP) on photographic film. The same method was used with a monoclonal rabbit anti-mouse IDO1 (CV152) and monoclonal antimouse β-tubulin antibody (1:1,000).

### Statistical Analysis

Data are expressed as the mean ± SEM. Differences between groups were analyzed by analysis of variance followed by the Bonferroni test. Differences between survival times were determined with the LogRank test. Data were analyzed using GraphPad Prism 6.2 software for Windows (GraphPad). A *p* value of ≤0.05 was considered statistically significant.

## Results

### IDO Inhibition Increases the Fungal Loads of DCs from Susceptible and Resistant Mice and Down Regulates NO, Kyn, and IDO mRNA Production

**Figure 1:**
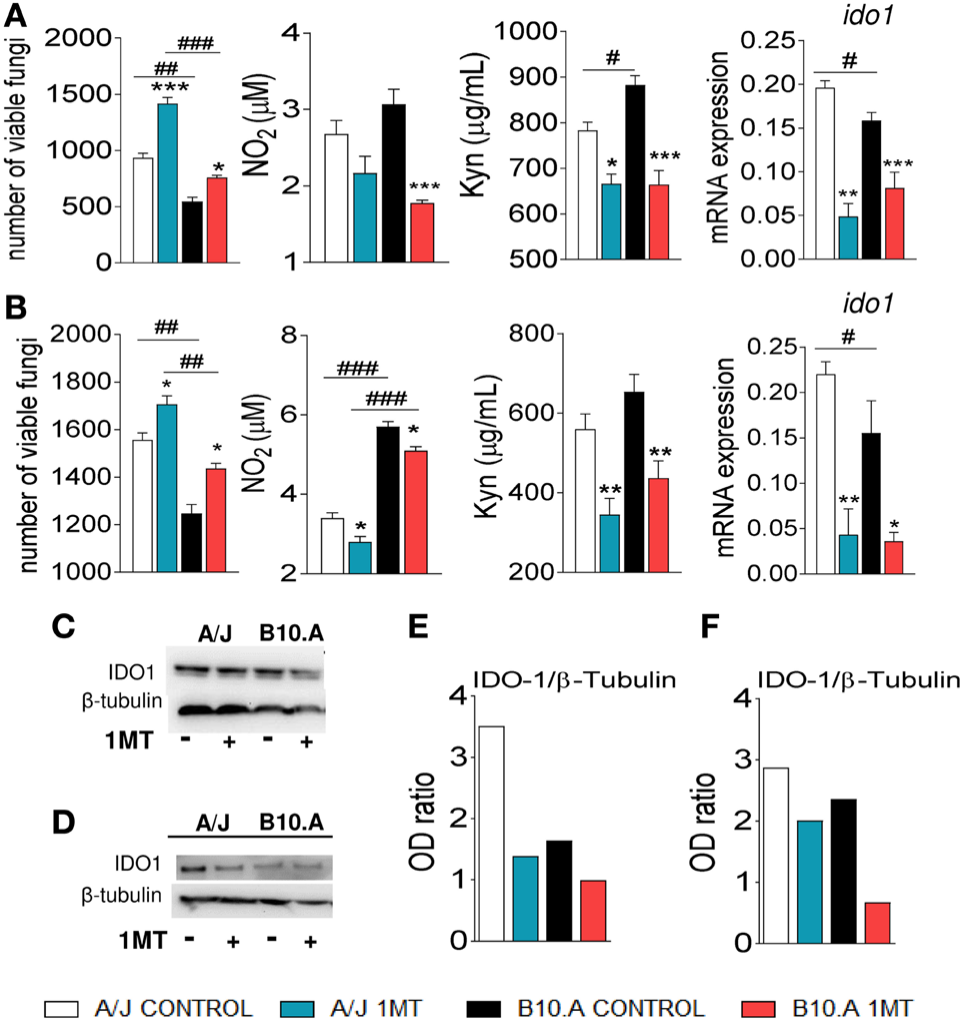
1-Methyl-dl-tryptophan (1MT) treatment reduces fungicidal activity, NO, kynurenine (Kyn), and indoleamine-2,3 dioxygenase (IDO) production by pulmonary dendritic cells (DCs) of *Paracoccidiodes Brasiliensis*-infected mice. Pulmonary DCs were obtained from 1MT treated or untreated A/J and B10.A mice at 96 h **(A,C,E)** and 2 weeks **(B,D,F)** after infection with 1 × 10^6^ yeasts and cultivated for 24 h at 37°C in 5% CO_2_. The cells were centrifuged, resuspended in 100 µL of culture medium and assayed for the presence of viable yeasts by a CFU assay. Supernatants from DCs cultures were used to determine the levels of nitrite and Kyn. In the same experimental conditions, DCs of A/J and B10.A mice were mixed with TRizol reagent for RNA extraction. IDO mRNA was measured using TaqMan real-time PCR assay. IDO was also characterized by immunoblotting. DCs were lysed, supernatants supplemented with a protease inhibitor and protein concentration determined by a BCA assay. After electrophoresis in polyacrylamide gel, proteins were transferred to nitrocellulose membranes and stained with anti-IDO antisera. Densitometry of bands was performed using ImageQuant TL 8.1 software. NO production was measured by Griess reagent, and Kyn were evaluated using Ehrlich’s reagent. The data represent the mean ± SEM of three independent experiments where the asterisk (*) represents a statistically significant difference between treatments (*p < 0.05, **p < 0.01, and ***p < 0.001) and the hashtag (#) represents the difference between the strains (^#^*p* < 0.05, ^##^*p* < 0.01, and ^###^*p* < 0.001).

Initially, we sought to investigate the effects of IDO inhibition on the interaction of DCs, from susceptible and resistant mice, with *P. brasiliensis*. To explore the effect of IDO inhibition *in vivo*, A/J and B10.A mice were treated or not with 1MT and subsequently subjected to i.t. infection with *P. brasiliensis* yeasts cells. Pulmonary DCs from both mouse strains were isolated 96 h and 2 weeks after infection and *in vitro* cultivated for 24 h. Using a CFU assay it was verified that in both postinfection periods 1MT-treated DCs presented increased fungal loads than untreated cells (Figures 1A,B). The levels of NO and Kyn in the supernatants of 1MT-treated DCs from both mouse strains was significantly lower than those detected in untreated groups (Figures 1A,B). In agreement with the reduced levels of Kyn observed, a reduced expression of IDO mRNA was found in 1MT-treated groups of both mouse strains (Figures 1A,B). The reduced IDO mRNA expression was further confirmed by the decreased protein production as detected by Western Blotting (Figures 1C–F).

### IDO Inhibition Promotes Increased Influx and Activation of Pulmonary DCs

**Figure 2:**
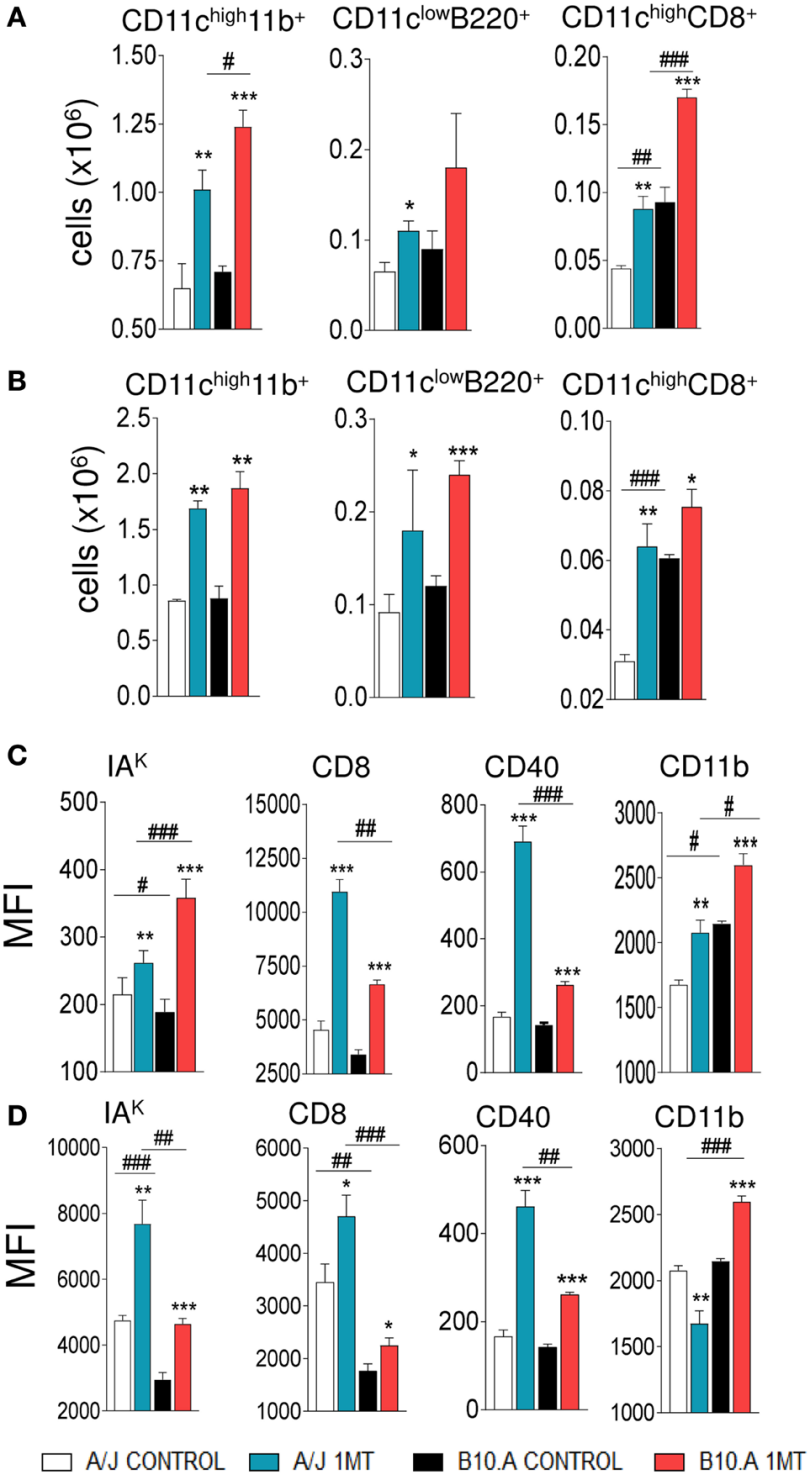
1-Methyl-dl-tryptophan (1MT) treatment increases the influx of activated dendritic cells (DCs) to the lungs. Number of myeloid (CD11c^high^11b^+^), plasmacytoid (CD11c^low^B220^+^), and lymphoid (CD11c^high^CD8^+^) DCs obtained 96 h **(A)** and 2 weeks **(B)** after infection of 1MT-treated and -untreated A/J and B10.A mice with 1 × 10^6^ viable *Paracoccidiodes brasiliensis* yeasts. Median fluorescence intensity (MFI) of IA^K^, CD86, CD11b, and CD40 expressed by DCs obtained from lungs of A/J and B10.A mice 96 h **(C)** and 2 weeks **(D)** after infection. DCs were obtained by fractionation with anti-CD11c magnetic beads and DCs phenotyping determined by flow cytometry. The data represent the mean ± SEM of three independent experiments where the asterisk (*) represents a statistically significant difference between treatments (*p < 0.05, **p < 0.01, and ***p < 0.001) and the hashtag (#) represents the difference between the strains (^#^*p* < 0.05, ^##^*p* < 0.01, and ^###^*p* < 0.001).

We have then asked whether the *in vivo* treatment with 1MT could interfere with the number and activation of pulmonary DCs. We verified that 96 h and 2 weeks after infection, 1MT-treated A/J and B10.A mice presented a higher number of myeloid, lymphoid and plasmacytoid DCs (CD11c^high^CD11b^+^, CD11c^high^CD8^+^, and CD11c^low^B220^+^, respectively) in their lungs than control mice (Figures 2A,B; Figure S1 in Supplementary Material). In addition, 1MT treatment increased the expression of MHC class II (IA^K^) and costimulatory molecules (CD86, CD40, CD11b) in pulmonary DCs obtained at both periods and mouse strains studied (Figures 2C,D). Thus, the increased fungal loads induced by 1MT treatment were associated with increased migration and activation of pulmonary DCs.

### IDO Inhibition Reduces the Secretion of DCs Cytokines

**Figure 3:**
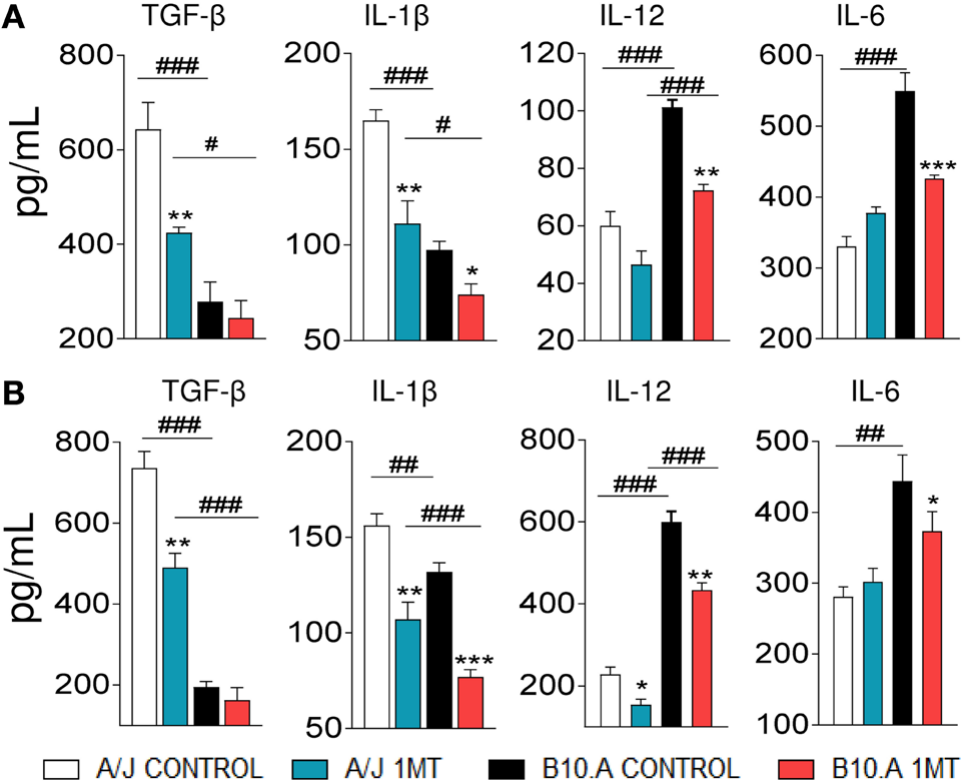
1-Methyl-dl-tryptophan (1MT) reduces the levels of TGF-β and IL-1β secreted by A/J dendritic cells (DCs), whereas in B10.A DCs 1MT reduces the secretion of IL-12, IL-6, and IL-1β. Pulmonary DCs were purified from lungs of 1MT treated and untreated A/J and B10.A mice 96 h **(A)** and 2 weeks **(B)** after i.t. infection with 1 × 10^6^ *Paracoccidiodes brasiliensis* yeasts cells. DCs were cultivated by 24 h, supernatants collected and analyzed for cytokine content (TGF-β, IL-1β, IL-12, and IL-6) by enzyme-linked immunosorbent assay (ELISA). The data represent the mean ± SEM of three independent determinations where the asterisk (*) represents a statistically significant difference between treatments (*p < 0.05, **p < 0.01, and ***p < 0.001) and the hashtag (#) represents the difference between the strains (^#^*p* < 0.05, ^##^*p* < 0.01, and ^###^*p* < 0.001).

To better evaluate the function of pulmonary DCs from 1MT-treated and untreated A/J and B10.A mice, the levels of cytokines present in the supernatants of cultivated cells were measured by ELISA. First, we could confirm our previous findings (28, 30) demonstrating that DCs from A/J mice produce higher levels of TGF-β and IL-1β than DCs from B10.A mice that, in contrast, are better producer of IL-12 and IL-6. We have found that 1MT treatment reduced the levels of TGF-β and IL-1β produced by A/J DCs at both postinfection periods. In contrast, reduced levels of IL-12, IL-6, and IL-1β were observed in 1MT-treated B10.A mice at both time points (Figures 3A,B).

### IDO Inhibition Reduces the Levels of Intracellular IDO and TGF-β in A/J DCs, whereas in B10.A DCs Diminishes the Levels of IDO, IL-12, and IL-6

**Figure 4:**
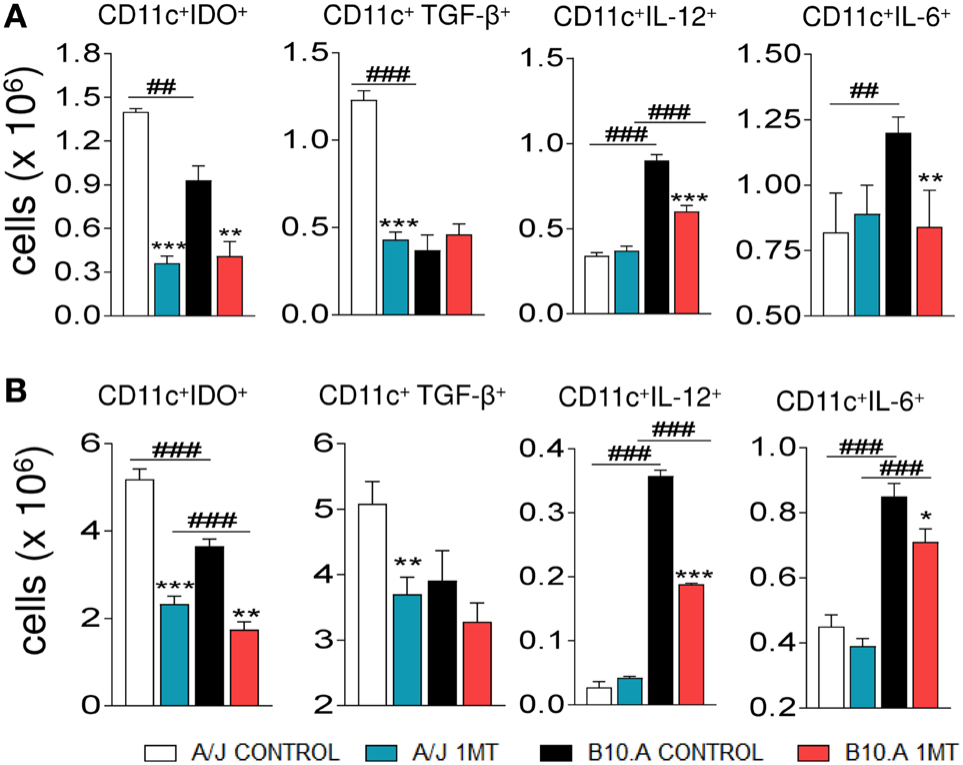
1-Methyl-dl-tryptophan (1MT) treatment reduces the number of TGF-β^+^ dendritic cells (DCs) in the lungs of A/J mice but reduces IL-12^+^ and IL-6^+^ DCs in B10.A mice. Number of pulmonary DCs expressing intracellular indoleamine-2,3 dioxygenase (IDO), TGF-β, IL-12, and IL-6, 96 h **(A)** and 2 weeks **(B)** after *Paracoccidiodes brasiliensis* infection. Lungs of 1MT treated and untreated A/J and B10.A-infected mice were excised and DCs cells purified by magnetic beads. The expression of intracellular cytokines was analyzed by flow cytometry. The data represent the mean ± SEM of three independent determinations where the asterisk (*) represents a statistically significant difference between treatments (*p < 0.05, **p < 0.01, and ***p < 0.001) and the hashtag (#) represents the difference between the strains (^##^*p* < 0.01 and ^###^*p* < 0.001).

We have also characterized the effect of IDO inhibition on intracellular cytokine (TGF-β, IL-12, and IL-6) content and IDO. Purified DCs from 1MT-treated and -untreated A/J and B10.A mice obtained at two postinfection periods were cultivated and intracellular proteins analyzed by flow cytometry. Compared with untreated controls, decreased numbers of TGF-β^+^ DCs were observed in the lungs of 1MT treated A/J mice. In contrast, reduced numbers of IL-12^+^ and IL-6^+^ DCs were detected in 1MT treated B10A mice (Figures 4A,B). In addition, reduced numbers of IDO positive DCs were observed in 1MT-treated DCs from both mouse strains and postinfection periods analyzed (Figures 4A,B).

### IDO Inhibition Enhances the Immunogenic Activity of DCs

Dendritic cells were isolated from 1MT-treated and untreated A/J and B10.A mice 96 h and 2 weeks after *P. brasiliensis* infection. In parallel, splenic lymphocytes from uninfected A/J and B10.A mice were obtained, labeled with CFSE, and cocultured with purified DCs. After 3 days, the proliferation of lymphocytes was analyzed by flow cytometry and the levels of IFN-γ and IL-2 were measured in culture supernatants.

**Figure 5:**
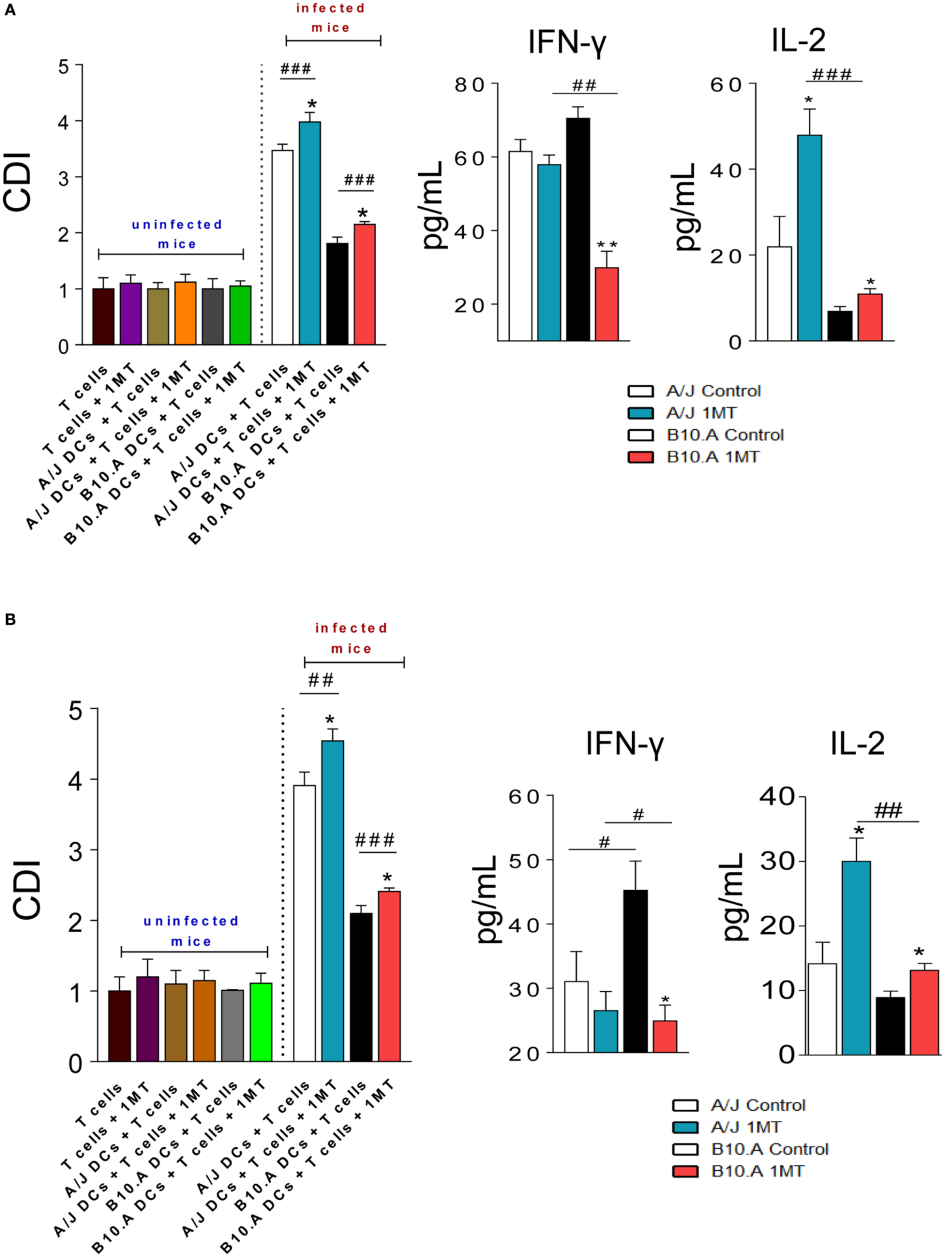
1-Methyl-dl-tryptophan (1MT) treatment increases the immunogenicity of dendritic cells (DCs). Pulmonary DCs were obtained from lungs of 1MT-treated and -untreated A/J and B10.A mice 96 h **(A)** and 2 weeks **(B)** after infection with 1 × 10^6^ *Paracoccidiodes brasiliensis* yeasts using anti-DC11c magnetic beads. Spleen lymphocytes from non-infected A/J and B10.A mice were obtained, labeled with CFSE (5 mM) and cocultivated with DCs, in a ratio of DC:lymphocyte of 1:10. In parallel, pulmonary DCs from uninfected A/J and B10.A mice were obtained using anti-DC11c magnetic beads and cultured with 1MT treated or untreated naive lymphocytes from non-infected A/J and B10.A mice. After 3 days of cocultivation, the cells were adjusted to 1 × 10^6^, labeled with specific anti-CD4 **(A)** and anti-CD8 **(B)** antibodies and analyzed by flow cytometry. The cell division index (CDI) was calculated as previously described by Mannering et al. (34) and was based on the number of CFSE^+^CD4^+^ or CFSE^+^CD8^+^ T cells found in the stimulated culture/number of CFSE^+^CD4^+^ or CFSE^+^CD8^+^ T cells in the unstimulated culture. The lymphocyte population was gated by FSC/SSC analysis. Fifty thousand cells were counted, and the data expressed as frequency of positive cells. Results are representative of three independent experiments. Cytokines were measured in culture supernatants by enzyme-linked immunosorbent assay (ELISA). The data represent the mean ± SEM of three independent determinations where the asterisk (*) represents a statistically significant difference between treatments (*p < 0.05 and **p < 0.01) and the hashtag (#) represents the difference between the strains (^##^*p* < 0.01 and ^###^*p* < 0.001).

As previously described with untreated pulmonary DCs (30), DCs from 1MT-treated and untreated A/J mice obtained at both postinfection periods induced higher lymphoproliferation than those of B10.A mice (Figures 5A,B). However, for both mouse strains at both periods assayed, the inhibition of IDO activity resulted in increased proliferation of lymphocytes. In cultures of 1MT treated or untreated naive lymphocytes no cell proliferation was observed. In the same way, no observe lymphoproliferation were detected when 1MT treated or untreated DCs from uninfected mice were used as APC. The levels of IFN-γ and IL-2 were measured in the supernatants of lymphoproliferation assays. Compared with untreated controls, decreased levels of IFN-γ were observed in 1MT-treated DCs from B10.A mice, whereas increased levels of IL-2 were detected in 1MT-treated DCs of both mouse strains and postinfection periods (Figures 5A,B).

### IDO Inhibition Expands CD4^+^ and CD8^+^ T Cells and Reduces Treg Cells

**Figure 6:**
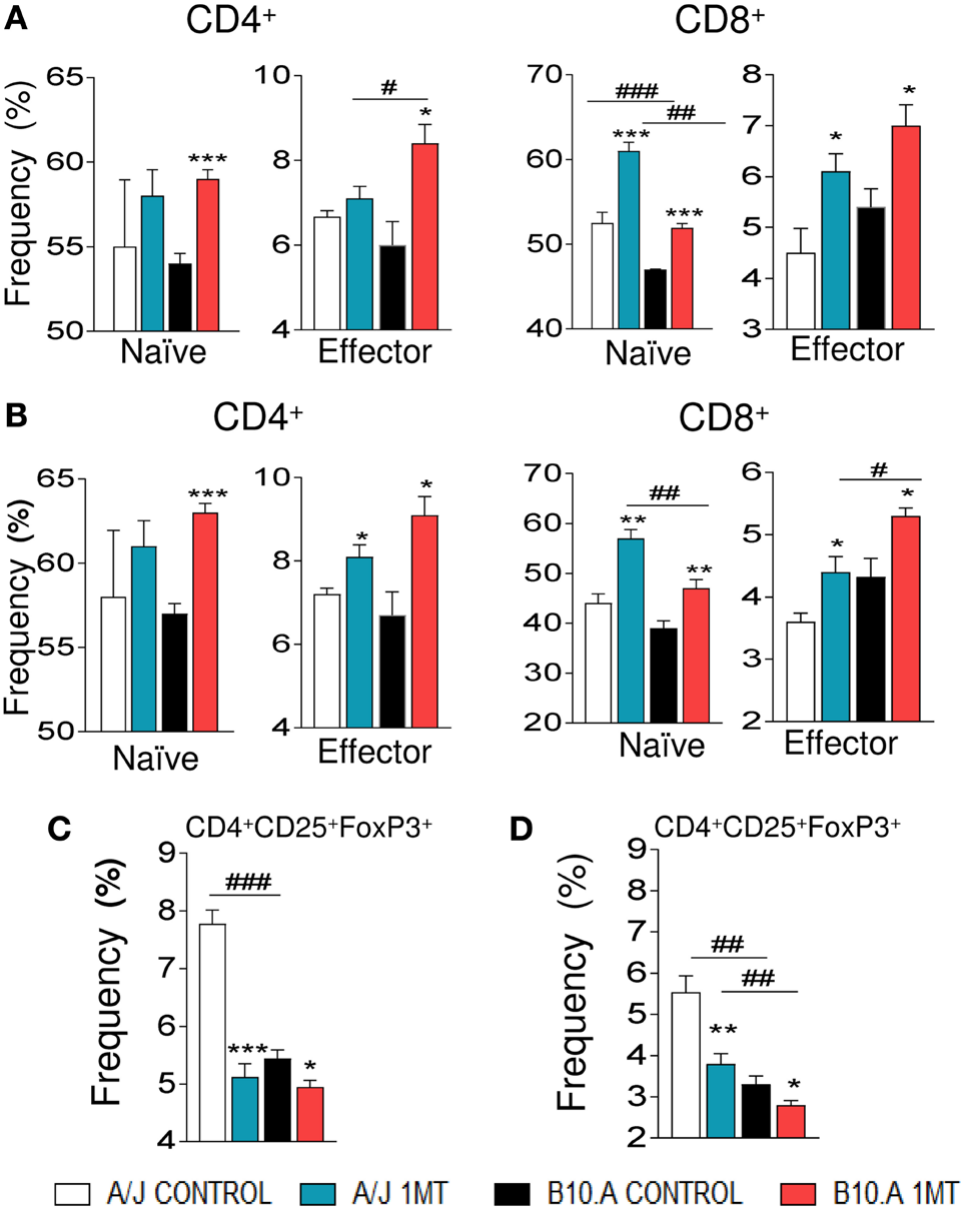
1-Methyl-dl-tryptophan (1MT) increases proliferation of CD4^+^ and CD8^+^ T cells and reduces regulatory T cells (Tregs). Spleen lymphocytes from uninfected A/J and B10.A mice were labeled with CFSE (5 mM) and cocultivated with purified dendritic cells (DCs) obtained from 1MT treated and untreated A/J and B10.A mice infected i.t. with 1 × 10^6^ of *Paracoccidiodes brasiliensis* yeasts. DCs were obtained at 96 h and 2 weeks postinfection, and cocultivated with CFSE labeled lymphocytes at a ratio of DC:lymphocyte of 1:10. After 3 days, the cells were adjusted to 1 × 10^6^, labeled with specific anti-CD4, -CD8, -CD44, -CD62L, -CD25, and -FoxP3 antibodies. The frequency of naive (CD62L^high^CD44^low^) and effector/memory (CD62L^low^CD44^high^) CD4^+^ and CD8^+^ T cells as well as CD4^+^CD25^+^FoxP3 regulatory T cells (Tregs) was measured by flow cytometry. Values are the mean of three independent experiments. The asterisks represent statistically significant differences between treatments (*p < 0.05, **p < 0.01, and ***p < 0.05). The hashtag marks represent statistically significant differences between strains (^#^*p* < 0.05, ^##^*p* < 0.01, ^###^*p* < 0.001).

Treatment of B10.A and A/J mice with 1MT increased the differentiation of T cells induced by isolated DCs. Indeed, increased frequency of naive CD4^+^ and CD8^+^ (CD4^+^CD44^low^CD62L^high^ and CD8^+^CD44^low^CD62L^high^, respectively) as well as memory/effector CD4^+^ and CD8^+^ (CD4^+^CD44^high^CD62L^low^ and CD8^+^CD44^high^CD62L^low^) T cells were detected when 1MT-treated DCs were cocultivated with naive lymphocytes (Figures 6A,B). Accordingly, reduced frequencies of Treg (CD4^+^CD25^+^FoxP3^+^) cells were observed when DCs were obtained from 1MT-treated resistant and susceptible mice (Figures 6C,D).

### IDO Is Regulated by IFN-γ in B10.A Mice and TGF-β in A/J Mice

Two opposite pathways were shown to regulate the production and activity of IDO. The IDO-IFN-γ axis is associated with the catalyst function of this enzyme, leads to trp starvation and Kyn production that in turn control pathogen growth and the inflammatory response. In contrast, the TGF-β-IDO axis has been linked with a signaling function of pIDO that promotes the differentiation of tolerogenic DCs and increases the expansion of Treg cells (12, 35, 36).

**Figure 7:**
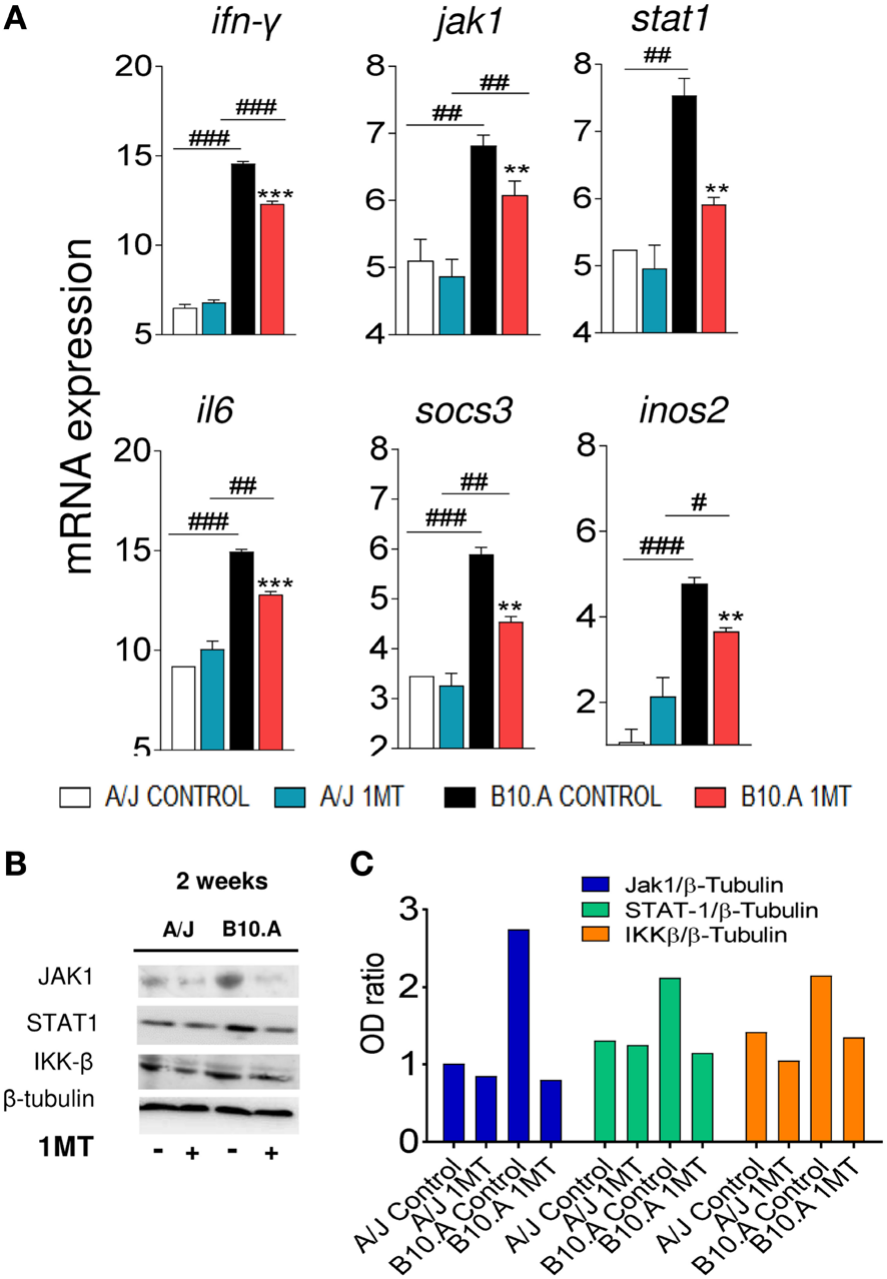
Indoleamine-2,3 dioxygenase (IDO) is regulated by IFN-γ signaling in B10.A dendritic cells (DCs). 1-Methyl-dl-tryptophan (1MT) treated or untreated B10.A and A/J mice were infected i.t. with 1 × 10^6^ yeast cells of *P. brasiliensis*, and 2 weeks after infection total lung inflammatory cells were obtained and DCs purified with anti-CD11c magnetic beads. **(A)** The relative expression of mRNA of IFN-γ, Janus kinase 1 (Jak1), signal transducer and activator of transcription (STAT1), IL-6, suppressor of cytokine signaling 3 (SOCS3), and nitric oxide synthase (NOS2) was measured by real-time PCR. **(B)** Presence of Jak1, Stat1, IKK-β, and IDO1 proteins was assessed by western blot in supernatants of lysed DCs. Proteins were estimated by analyzing the intensity of each band normalized by β-tubulin, used as control. **(C)** Densitometry of bands was performed using ImageQuant TL 8.1 software. The asterisks represent statistically significant differences between treatments (*p < 0.05, **p < 0.01, ***p < 0.001). The hash marks represent statistically significant differences between strains (^#^*p* < 0.05, ^##^*p* < 0.01, ^###^*p* < 0.001).

**Figure 8:**
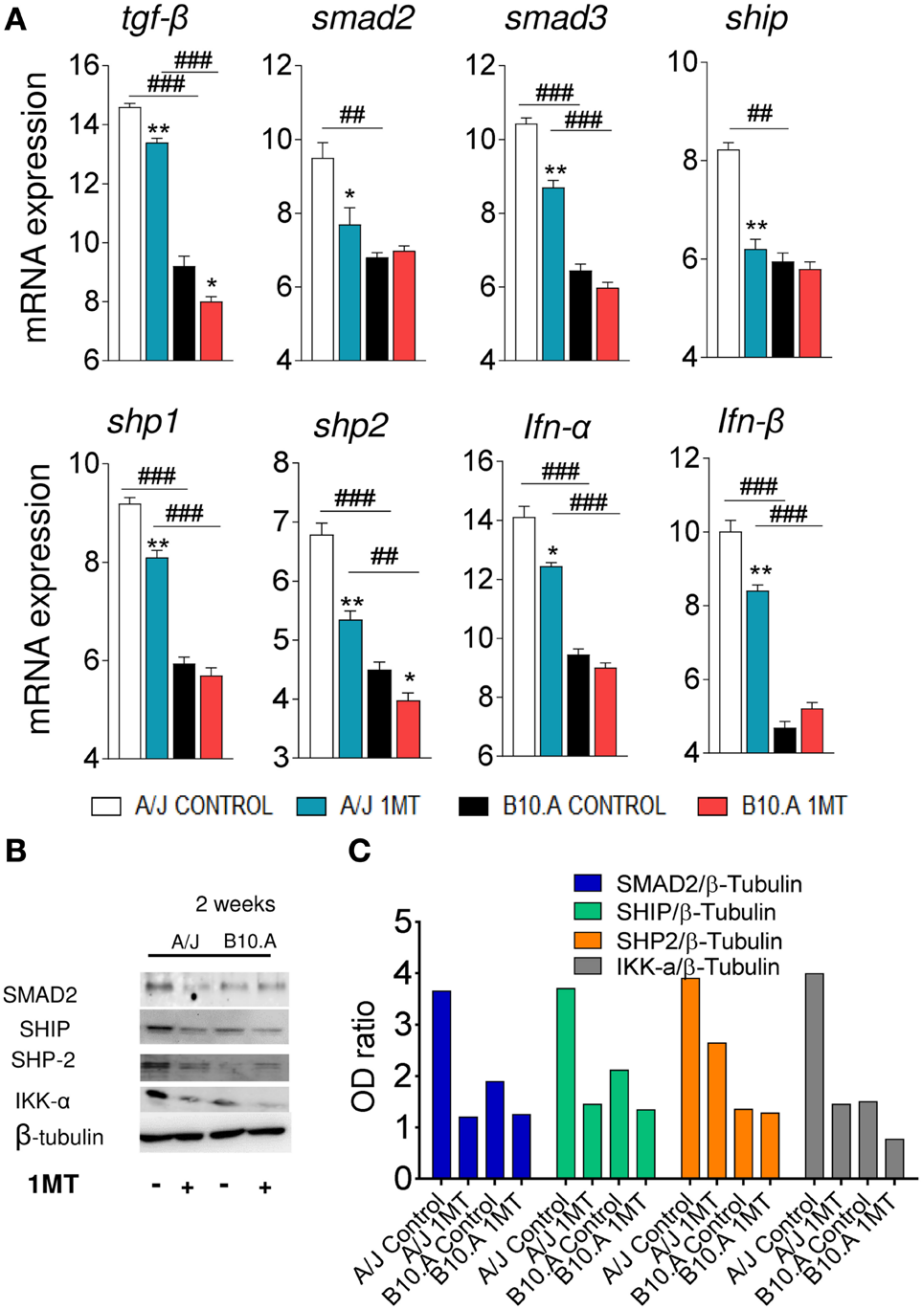
Indoleamine-2,3 dioxygenase (IDO) is regulated by TGF-β signaling in A/J dendritic cells (DCs). 1-Methyl-dl-tryptophan (1MT)-treated or -untreated B10.A and A/J mice were infected i.t. with 1 × 10^6^ *Paracoccidiodes brasiliensis* yeasts and 2 weeks after infection total lung inflammatory cells were obtained and DCs purified by anti-CD11c magnetic beads. **(A)** The relative expression of mRNA of TGF-β, Smad2, Smad 3, SHIP, SHP-1, SHP-2, IFN-α, and IFN-β was measured by real-time PCR. **(B)** Smad2, Ship, Shp-2, IKK-α and IDO1, and pIDO protein expression was assessed by western blot in supernatants of lysed DCs. **(C)** Proteins were estimated by analyzing the intensity of each band normalized by β-tubulin, used as control. Densitometry of bands was performed using ImageQuant TL 8.1 software. Values are the mean ± SEM of three independent experiments; the asterisks represent statistically significant differences between treatments (*p < 0.001, **p < 0.01, ***p < 0.05). The hash marks represent statistically significant differences between strains (^#^*p* < 0.05, ^##^*p* < 0.01, ^###^*p* < 0.001).

We speculated that in B10.A mice IDO is mainly catalyst and IFN-γ-regulated, whereas in A/J mice IDO has a predominant signaling function induced by TGF-β. To explore this hypothesis, the expression of genes and proteins related to IFN-γ and TGF-β signaling pathways was characterized in DCs of 1MT-treated and untreated B10.A and A/J mice obtained at 96 h (Figures S3 and S4 in Supplementary Material) and 2 weeks of infection. As shown in Figure 7A, DCs from B10.A mice expressed higher levels of mRNA from genes associated with IFN-γ signaling. Therefore, 1MT treatment led to a marked reduction in the expression of IFN-γ, Jak1, Stat1, IL-6, SOCS3, and NOS2 mRNA by DCs from B10.A mice but had a minor effect on DCs of A/J mice (Figure 7A). These findings indicate that in B10.A mice the IDO function is mainly catalyst and regulated by IFN-γ. Immunoblotting assays confirmed these results, and increased amounts of JAK1, STAT-1, and IKK-β proteins were found in DCs extracts of B10.A DCs (Figures 7B,C). In contrast to B10.A mice, DCs from A/J mice express higher levels of mRNA from genes associated with TGF-β signaling (Figure 8). Compared with B10.A DCs, cells from A/J mice expressed increased levels of TGF-β, SMAD2, SMAD3, SHIP1, SHP1, SHP2, IFN-α, and IFN-β mRNA. 1MT treatment significantly reduced these levels in A/J DCs but had no effect on B10.A cells (Figure 8A). Assessing protein production, increased amounts of SMAD2, SHIP1, SHP2, and IKK-α were observed in extracts of A/J DCs (Figures 8B,C).

### A/J Mice pIDO and Use the Non-Canonical Pathway of NF-κB Activation, whereas B10.A Mice Express Non-pIDO and Use the Canonical Pathway of NF-κB Activation

**Figure 9:**
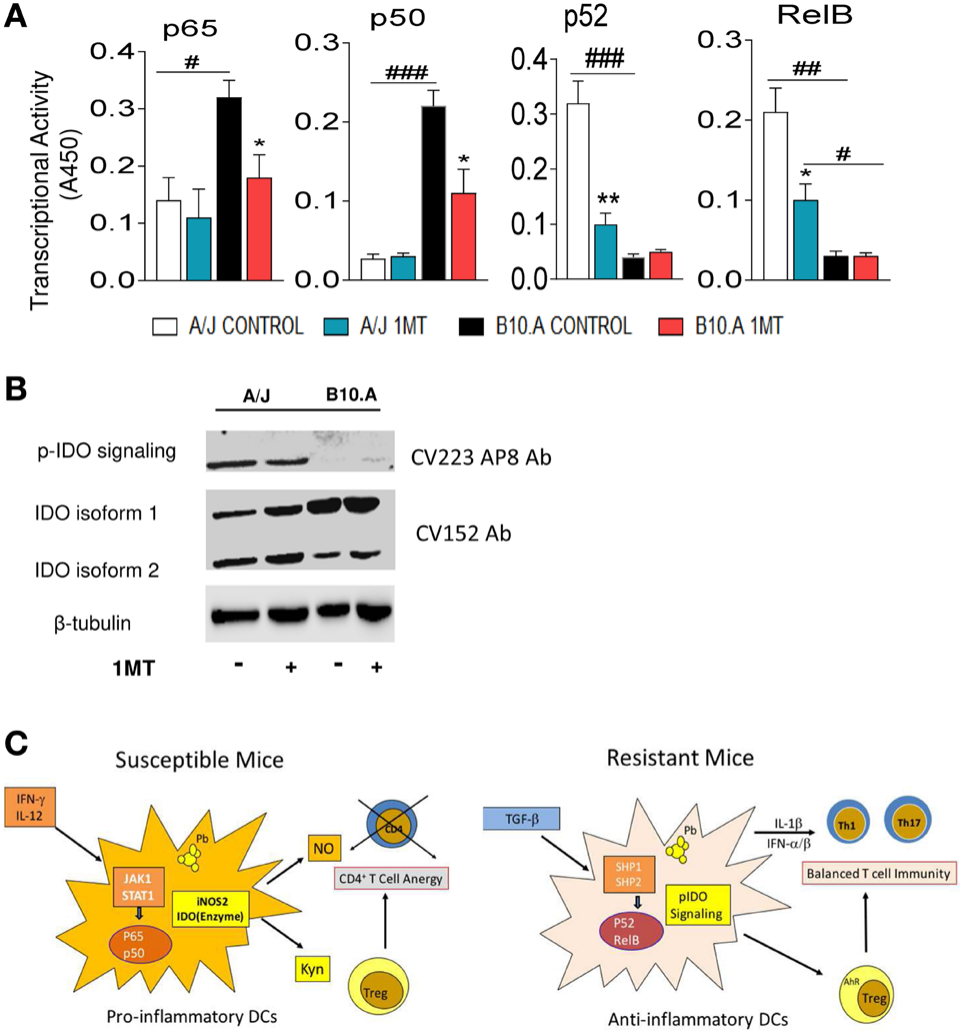
Phosphorylated indoleamine-2,3 dioxygenase (pIDO) of A/J mice activate the non-canonical pathway of NF-κB, whereas in B10.A dendritic cells (DCs) non-phosphorylated IDO use the canonical pathway of NF-κB activation. **(A,B)** Quantitation of p65, p50, p52, and RelB by enzyme-linked immunosorbent assay (ELISA) in nuclear extracts of DCs. Results are presented as absorbance at 450 nm (A450). Values are the mean ± SE of three independent experiments. The asterisks represent statistically significant differences between treatments (*p < 0.05, **p < 0.01, ***p < 0.001). The hash marks represent statistically significant differences between strains (^#^*p* < 0.05, ^##^*p* < 0.01, ^###^*p* < 0.001). **(C)** Expression of IDO and phosphorylated IDO was assessed by Western blot in lung homogenates of 1MT treated and untreated B10.A and A/J mice i.t. infected with 1 × 10^6^ *Paracoccidiodes brasiliensis* yeasts. pIDO was revealed by a specific antibody that recognizes the phosphorylated ITIM moiety of IDO (CV223 AP8) and the enzyme IDO by a specific monoclonal antibody (CV152). **(D)** In susceptible B10.A mice, IDO is induced by IFN-γ that activates the canonical pathway of NF-κB and enhanced iNOS and IDO expression resulting in anergy of T cell immunity. In resistant A/J mice, TGF-β signaling induces the non-canonical pathway of NF-κB activation and continuous synthesis of TGF-β that confers an stable tolerogenic behavior to DCs that control immunity and tissue pathology.

Because IFN-γ signaling is mainly associated with the canonical pathway of NF-κB activation, the transcriptional activity of p65 and p50 were measured by ELISA in the nuclear extracts of 1MT-treated and -untreated DCs. As depicted in Figure 9A, DCs from B10.A mice showed increased expression of nuclear p65 and p50 than those of A/J mice. Furthermore, 1MT treatment reduced this production by B10.A DCs (Figure 9A). An equivalent result was observed with DCs obtained at 96 h of infection (Figure S1 in Supplementary Material). The prevalent non-canonical activation of NF-κB by A/J DCs was confirmed by the increased levels of nuclear p52 and RelB produced in comparison with B10.A DCs (Figure 9A). Treatment with 1MT caused a significant reduction of p52 and RelB only in A/J DCs. Again, an equivalent result was observed in pulmonary DCs obtained at 96 h of infection (Figure S2 in Supplementary Material). To further confirm the signaling function of IDO, lung extracts from 1MT treated and untreated B10.A and A/J mice were obtained 2 weeks after infection and the expression of pIDO was assessed by immunoblotting using a specific mAb (CV223 AP8 Ab) that recognizes the phosphorylated moiety of ITIM. As depicted in Figure 9B, only A/J mice express pIDO and 1MT treatment did not affect the production of this molecule. Using a polyclonal antibody to IDO (CV152 Ab) two isoforms of IDO, and the elevated expression of the isoform 1 by B10.A DCs were seen. The Figure 9C summarizes the main findings obtained in this study. In susceptible B10.A mice IDO is induced by IFN-γ that activates the canonical pathway of NF-κB and enhanced iNOS and IDO expression resulting in anergy of T cell immunity. In resistant A/J mice, TGF-β signaling induces the non-canonical pathway of NF-κB activation and continuous synthesis of TGF-β that confers an stable tolerogenic behavior to DCs that control immunity and tissue pathology.

## Discussion

The antimicrobial and immunoregulatory functions of IDO have been recognized as central mechanisms used by infected hosts to control pathogen growth and excessive inflammatory responses (37, 38). Both IDO activities were used by resistant and susceptible mice to control pulmonary PCM. However, only in susceptible mice IDO inhibition caused a sustained fungal growth, exacerbated tissue pathology and increased mortality rates. This is the worst scenario for host fitness because it allies uncontrolled pathogen growth with exacerbated, but inefficient, inflammation resulting in warful tissue destruction. These findings led us to further investigate the role of IDO in the resistance mechanisms to pulmonary PCM. We found the IDO inhibitor 1-MT as two stereoisomers, 1-D-MT and 1-L-MT. Most of the studies using the IDO enzyme model employed the racemic mixture 1-DL-MT to inhibit the enzime, independent of the known IDO isoforms, IDO1 and IDO2, as we did in this work. Indeed, recent studies have shown that IDO1 is the preferentially inhibited by1-L-MT, while 1-D-MT inhibits IDO2 (39, 40). These findings directed our subsequent studies on the inhibition of IDO using deficient mice, as we have shown in recent work with IDO1-deficient (IDO1^−/−^) C57BL/6 mice (41). Using isolated pulmonary DCs, we verified that IDO inhibition led to increased recovery of yeasts from both A/J and B10.A cells. This finding could be attributed to the increased trp availability (42–44), but also to the reduced NO production, an important fungicidal mechanism of macrophages and DCs (26, 27, 30).

Interestingly, IDO inhibition by 1MT reduced the typical cytokines that characterize the innate response of B10.A and A/J mice (26, 27, 30). Furthermore, the assessment of intracellular cytokines has further emphasized the higher expression of IDO and TGF-β by A/J DCs, whereas the proinflammatory cytokines IL-12 and IL-6 were more expressed by B10.A DCs. This led us to suppose that IDO activity in B10.A mice was mainly catalytic, whereas in A/J mice was predominantly signaling and involved in signal transduction (12). These investigators clearly demonstrated that TGF-β signaling activates the tyrosine kinase Fyn that phosphorylates ITIM domains of IDO. This promotes the association of IDO with tyrosine phosphatases (SHP1 and SHP2) that results in the activation of the non-canonical NF-κB pathway (p52/RelB), which translocates to the nucleus and induces the production of type I IFNs (IFNα/β), TGF-β, and IDO (12).

An increased number of activated DCs with enhanced immunogenic activity was detected in the lungs of 1MT treated mice. The increased proliferation of naive lymphocytes was concomitant with increased levels of IL-2 in A/J cocultures and decreased IFN-γ production by B10.A cells mirroring the main suppressive mechanisms developed by susceptible and resistant mice (25, 26, 30, 45). This enhanced immunogenic activity was further confirmed by the increased proliferation of naive and effector/memory CD8^+^ and CD4^+^ T cells concomitant with a reduced expansion of CD4^+^CD25^+^Foxp3^+^ Treg cells, a finding that may be also associated with the reduced levels of suppressive Kyn produced (46, 47). This tolerogenic function of Kyn on T cells has been linked to the activation of the AhR, a ligand activated transcription factor (46, 48) that participates in the pIDO-tolerogenic DCs-Treg loop of immunoregulation.

Further assessing the mechanisms involved in the divergent activities of IDO, a preferential IFN-γ signaling pathway was observed in B10.A DCs which expressed increased levels of IFN-γ and Jak1-Stat1 mRNA. In addition, elevated expression of IL-6, SOCS3, and iNOS2 were also observed indicating a prevalent IFN-γ-mediated catalytic activity. These data were further confirmed by the increased synthesis of Stat1 and IKKβ proteins involved in IFN-γ signaling. The striking expression of IL-6 and SOCS3 suggests that in susceptible mice IDO degradation may occur *via* proteasomal activity (49). Our data have also confirmed the prevalent TGF-β-mediated activation of A/J DCs which express high levels of TGF-β, Smad2, Smad3, Ship1, Shp1, Shp2, IFN-α, and IFN-β mRNA. The high levels of IKK-α, Smad2, Ship1, and Sph-2 proteins expressed by these cells have further confirmed the use of the TGF-β signaling by DCs from resistant mice. These data agree with the signaling cascade that promotes a tolerogenic activity in plasmacytoid DCs previously described (12) and explain the continuous production of TGF-β by A/J mice that favors the conversion of naive CD4^+^ T cells into Treg cells (25). Interestingly, the inhibition of IDO by 1MT showed little interference with the TGF-β-mediated pathway used by A/J DCs, demonstrating that the signaling function of the IDO has predominance over the catalytic activity.

The predominant expression of p65 and p50 by B10.A DCs and the higher levels of p52 and RelB by A/J DCs showed that B10.A and A/J mice predominantly use, respectively, the canonical and non-canonical pathways of NF-κB activation by their pulmonary DCs. More importantly, we could demonstrate the presence of pIDO in the lungs of A/J mice, whereas the non-phosphorylated forms of IDO were observed in B10.A mice. Therefore, we could clearly demonstrate that in B10.A mice IDO has a catalytic function, whereas in A/J mice, besides its enzymatic activity, IDO has a signaling function.

Similarly with other infection models, our studies in experimental PCM showed that the balanced activation of the immune system is fundamental for the protection of hosts, and the excessive activation of pro- or anti-inflammatory mechanisms leads to severe disease (23, 29, 50, 51). In susceptible mice, the suppression of adaptive immunity is a consequence of excessive proinflammatory activity of innate immunity, which, however, is able to control the initial fungal burden. In resistant mice, in contrast, an anti-inflammatory, TGF-β dominated activity is observed impairing the initial control of fungal growth. This activity, however, is balanced by the marked dectin-1 and NLRP3 activation and synthesis of TNF-α and IL-1β by A/J macrophages. This pattern of response leads to the subsequent differentiation of Th1/Th17 mixed responses, always strongly controlled by elevated numbers and activity of Treg cells that control tissue pathology (24–27, 29). This is an unusual but interesting model of resistance and susceptibility to a primary fungal pathogen not previously described.

Host defenses are governed by resistance and tolerance mechanisms, the first involved in reducing pathogen burden during infection and the latter associated with mechanisms that protected the hosts from pathogen- or immune-induced damage. These mechanisms are not mutually exclusive and both can contribute to host fitness (52, 53). Our studies clearly demonstrate that the resistance mechanisms used by A/J mice are governed by resistance to disease and not resistance to pathogen growth. Although not intensely explored in infectious diseases, tolerance to disease is well known and described in plant pathology where host protection is not achieved by the elimination of the pathogen, but it is based on the control of host fitness. This response allows pathogen persistence, but its excessive growth is partially controlled by immune mechanisms that are tightly regulated to preserve homeostasis (52). This is also observed in the host responses to commensal fungi such as *Aspergillus fumigatus* and *Candida albicans* that concomitantly control fungal burden and tissue inflammation (50). The important influence of the IDO-AhR-Treg-Th17 axis on the control of this well-balanced response to commensal microorganisms has been well described in the literature (44, 54, 55). The IFN-γ-mediated activation of IDO and Kyn synthesis by tissue DCs confers a tolerogenic profile to these cells that promote the differentiation of naive T cells into Foxp3^+^ Treg cells *via* activation of AhR that prevents the excessive expansion of Th17 lymphocytes (44, 56). This tightly regulated immunity confers immune homeostasis that results in microbial resistance but avoids tissue pathology. The signaling property of pIDO induced by TGF-β activation of DCs was more recently described and has been viewed as potent immunoregulatory mechanism that confer stable tolerogenic activity to plasmacytoid DCs (12, 36). This tolerogenic mechanism was validated in an *in vivo* model of delayed-type hypersensitivity response and in the protective activity of endotoxin-tolerant state of mice against Gram-positive and Gram-negative infections (12, 54). However, to our knowledge, this mechanism of disease tolerance was never described in the host responses to primary fungal pathogens. Indeed, pIDO-mediated tolerance was poorly explored in infectious pathologies. Therefore, we believe that the findings here described open new perspectives to understand resistance against PCM and other primary fungal pathogens where excessive inflammation has been associated with severe disease (57, 58). Previous results obtained in our experimental model, not well understood when originally obtained, can now be re-evaluated in face of the IDO mediated mechanism here described. The subcutaneous immunization with viable yeasts confers sterile immunity to i.p. challenged B10.A mice but partial immunoprotection in A/J mice that remain with persistent fungal loads (59, 60). We have also previously described that *P. brasiliensis* infection induces an elevated production of TGF-β and M2 differentiation of A/J macrophages which express high levels of arginase (27), another finding which is not in line with the mechanisms of pathogen resistance usually described. However, this finding may now be better appreciated and understood because arginase expression by innate immune cells was reported to be essential for the differentiation of tolerogenic DCs, whose signaling activity is mediated by pIDO (61).

In conclusion, the studies here reported clearly demonstrate that susceptibility to PCM is mainly mediated by the proinflammatory IFN-γ-IDO axis of innate responses, whereas resistance relies on the initial anti-inflammatory TGF-β-pIDO-Treg axis that confers a sustained tolerogenic phenotype in pulmonary DCs allowing A/J mice to interact with a primary fungal pathogen as a commensal microbe. Therefore, the signaling function of pIDO leads to an efficient fungus–-host interaction that conditions advantages to both partners, and may be explored in further innovative anti-fungal therapy.

## Ethics Statement

The experiments were performed in strict accordance with the Brazilian Federal Law 11,794 establishing procedures for the scientific use of animals, and the State Law establishing the Animal Protection Code of the State of São Paulo. All efforts were made to minimize animal suffering. The procedures were approved by the Ethics Committee on Animal Experiments of the Institute of Biomedical Sciences of University of São Paulo (Proc.180/11/CEEA).

## Author Contributions

EA designed, conducted, and analyzed all experiments. FL, CF, and TC performed and analyzed experiments. CV characterized IDO and IDO phosphorylation. LR provided conceptual help and wrote the article. PP provided conceptual help and precious reagents for IDO analysis. VC planned experiments, supervised the overall study, and wrote the manuscript.

## Conflict of Interest Statement

The authors declare that the research was conducted in the absence of any commercial or financial relationships that could be construed as a potential conflict of interest.
