# Supplementary material for: Disease Tolerance Mediated by Phosphorylated Indoleamine-2,3 Dioxygenase Confers Resistance to a Primary Fungal Pathogen
Source: Front Immunol. 2017 Nov 13;8:1522. doi: 10.3389/fimmu.2017.01522 (PMC5693877; doi:10.3389/fimmu.2017.01522)
Supplement: Supplementary file 1 [file image_1.pdf]

Supplem. Figure-1

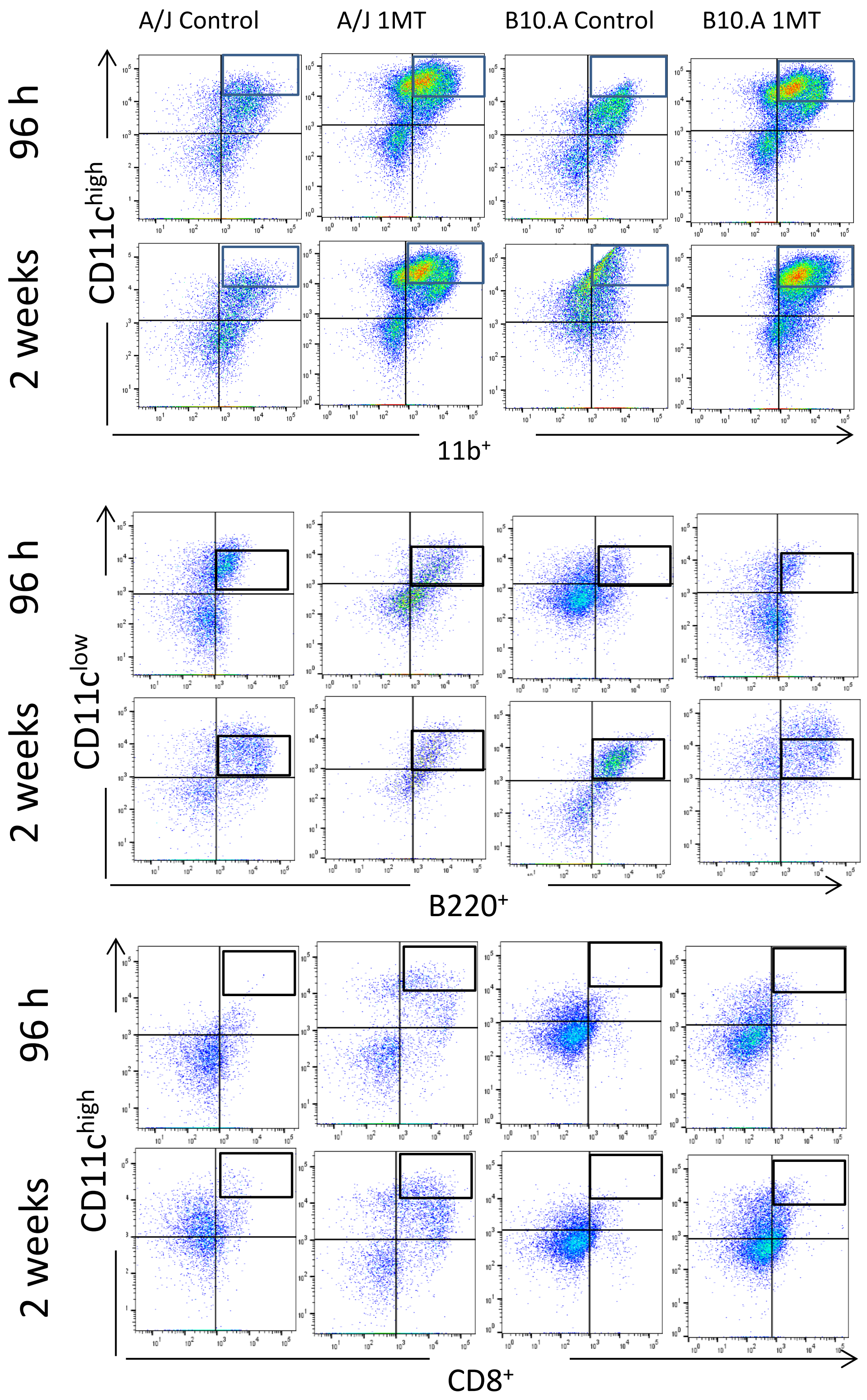

Supplementary Figure 1. Gate strategy to determine DC cell subpopulations by flow cytometry. Characterization of myeloid (**CD11c^{high}11b^{+}**), plasmacytoid (**CD11c^{low}B220^{+}**) and lymphoid (**CD11c^{high}CD8^{+}**) DCs by flow cytometry.
