# Supplementary material for: Disease Tolerance Mediated by Phosphorylated Indoleamine-2,3 Dioxygenase Confers Resistance to a Primary Fungal Pathogen
Source: Front Immunol. 2017 Nov 13;8:1522. doi: 10.3389/fimmu.2017.01522 (PMC5693877; doi:10.3389/fimmu.2017.01522)
Supplement: Supplementary file 2 [file image_2.pdf]

Supplem. Figure-2

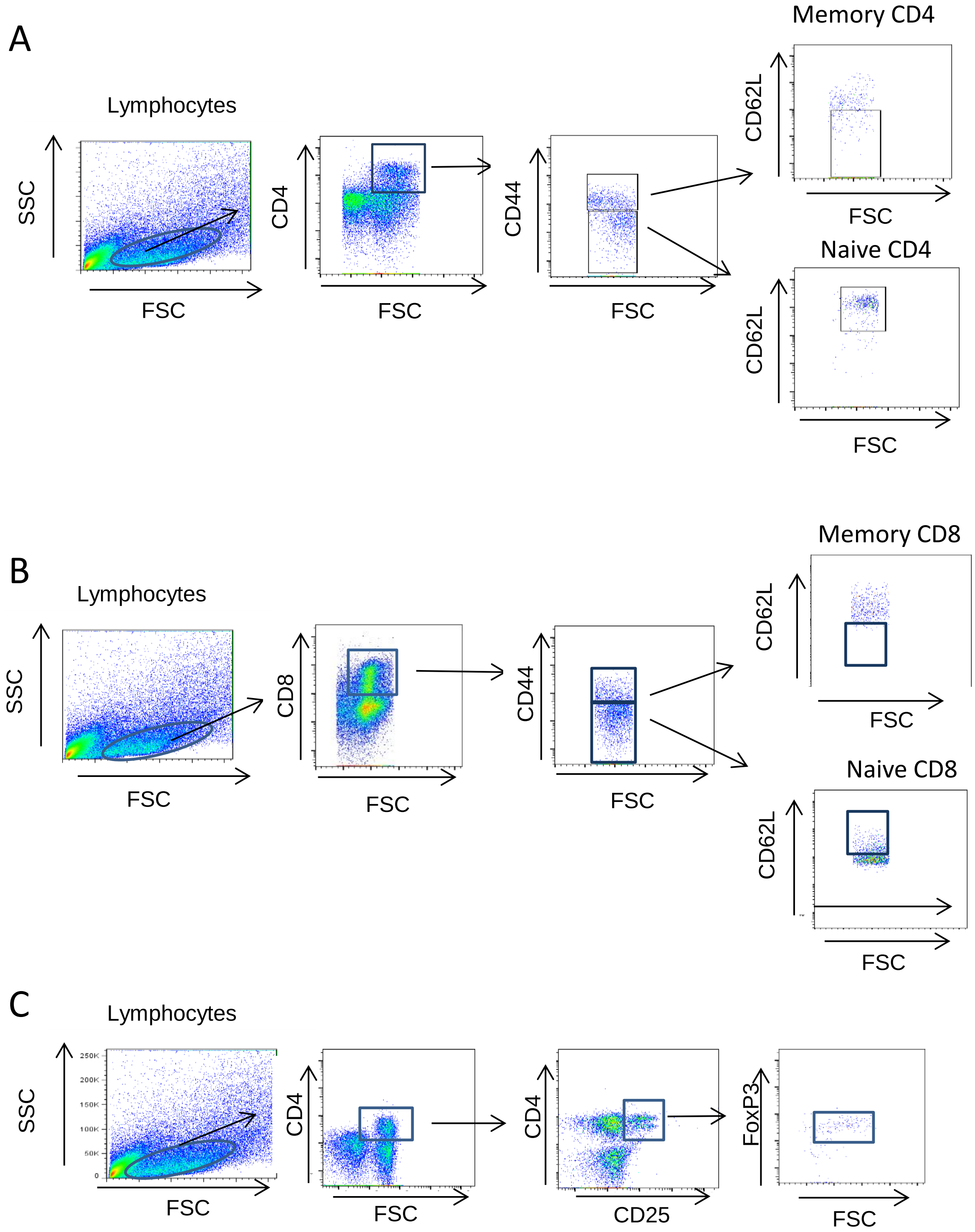

Supplementary Figure 2. Gate strategy to determine T cell subpopulations by flow cytometry. (A) Characterization of naïve ($CD62L^{high}CD44^{low}$), (B) effector/memory ($CD62L^{low}CD44^{high}$) $CD4^{+}$ and $CD8^{+}$ T cells, and (C) $CD4^{+}CD25^{+}$FoxP3 Treg cells by flow cytometry.
