# Supplementary material for: Disease Tolerance Mediated by Phosphorylated Indoleamine-2,3 Dioxygenase Confers Resistance to a Primary Fungal Pathogen
Source: Front Immunol. 2017 Nov 13;8:1522. doi: 10.3389/fimmu.2017.01522 (PMC5693877; doi:10.3389/fimmu.2017.01522)
Supplement: Supplementary file 4 [file image_4.pdf]

Supplem. Figure-4

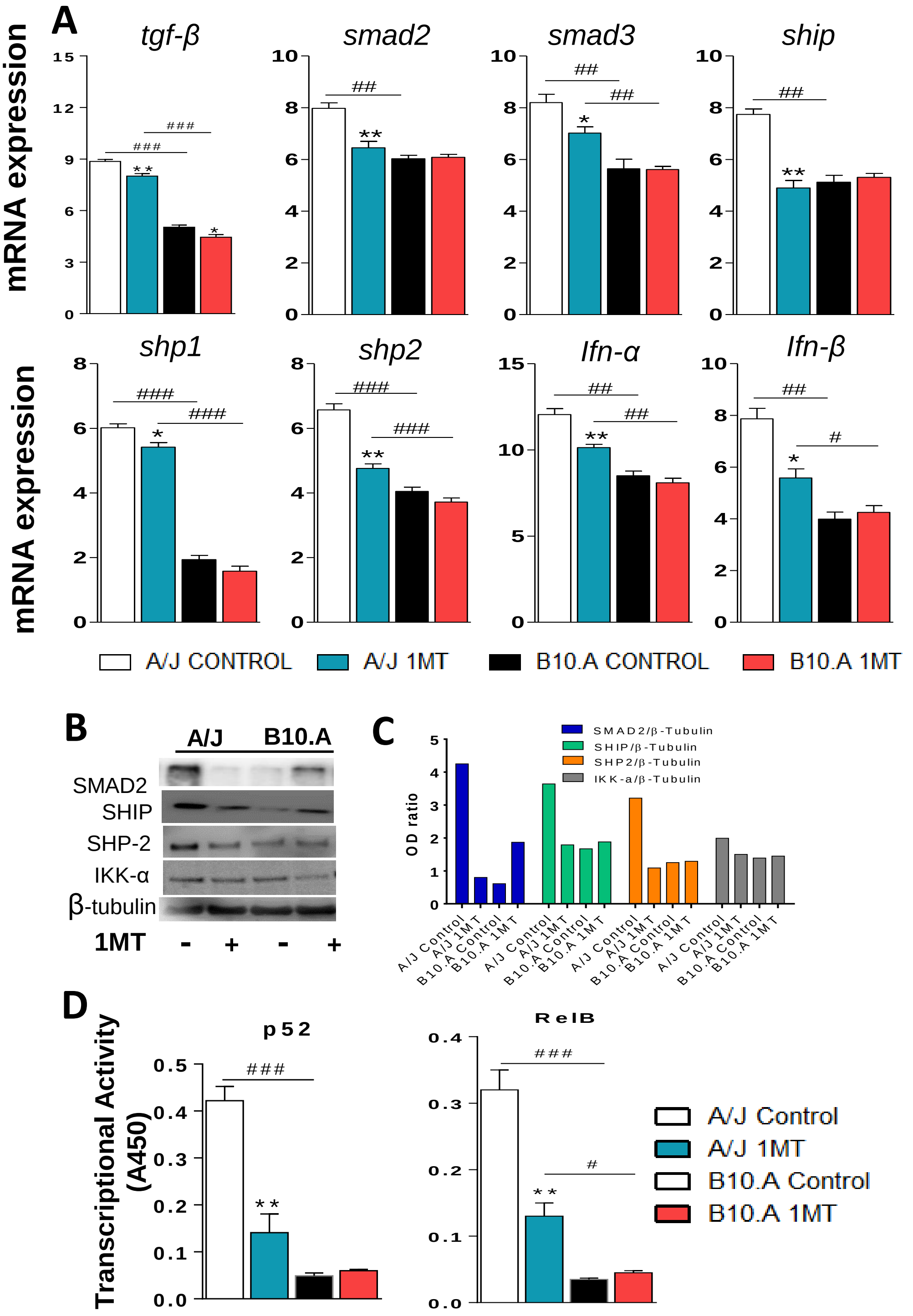

Supplementary Figure 4. **In A/J mice, IDO is mainly regulated by TGF-β signaling.** 1MT treated or untreated B10.A and A/J mice were infected i.t. with 1x10^6 *P. brasiliensis* yeasts and 96 h weeks after infection total lung inflammatory cells were obtained and DCs purified by anti-CD11c magnetic beads. (A) The relative expression of mRNA of TGF-β, Smad2, Smad 3, SHIP, SHP-1, SHP-2, IFN-α and IFN-β was measured by real-time PCR. (B) Smad2, Ship, Shp-2, IKK-α and IDO1, and pIDO protein expression was assessed by western blot in supernatants of lysed DCs. (C) Proteins were estimated by analyzing the intensity of each band normalized by β-tubulin, used as control. Densitometry of bands was performed using ImageQuant TL 8.1 software. (D) Quantitation of p65, p50, p52 and RelB by ELISA in nuclear extracts of DCs. Results are presented as absorbance at 450 nm (A450). Values are the mean ± SEM of three independent experiments; the asterisks represent statistically significant differences between treatments (**p* <0.001, ** *p* < 0.01, ***P <0.05). The hash marks represent statistically significant differences between strains (#*p* <0.05, ## *p* < 0.01, ### *p* <0.001).
